# Three‐dimensional microtissues essentially contribute to preclinical validations of therapeutic targets in breast cancer

**DOI:** 10.1002/cam4.630

**Published:** 2016-01-14

**Authors:** Natalie Falkenberg, Ines Höfig, Michael Rosemann, Justine Szumielewski, Sabine Richter, Kenji Schorpp, Kamyar Hadian, Michaela Aubele, Michael J. Atkinson, Nataša Anastasov

**Affiliations:** ^1^ Institute of PathologyHelmholtz Center MunichGerman Research Center for Environmental HealthIngolstaedter Landstrasse 185764NeuherbergGermany; ^2^ Institute of Radiation BiologyHelmholtz Center MunichGerman Research Center for Environmental HealthIngolstaedter Landstrasse 185764NeuherbergGermany; ^3^ Assay Development and Screening PlatformInstitute of Molecular Toxicology and PharmacologyHelmholtz Center MunichGerman Research Center for Environmental HealthIngolstaedter Landstrasse 185764NeuherbergGermany; ^4^ Radiation BiologyTechnical University of MunichIsmaninger Strasse 2281675MunichGermany

**Keywords:** 3D microtissue, combination, HER2 knockdown, model, mouse xenografts, radiation, spheroid, trastuzumab

## Abstract

A 3D microtissues using T47D and JIMT‐1 cells were generated to analyze tissue‐like response of breast cancer cells after combined human epidermal growth factor receptor 2 (HER2)‐targeted treatment and radiation. Following lentiviral knockdown of HER2, we compared growth rate alterations using 2D monolayers, 3D microtissues, and mouse xenografts. Additionally, to model combined therapeutic strategies, we treated HER2‐depleted T47D cells and 3D microtissues using trastuzumab (anti‐HER2 antibody) in combination with irradiation. Comparison of HER2 knockdown with corresponding controls revealed growth impairment due to HER2 knockdown in T47D 2D monolayers, 3D microtissues, and xenografts (after 2, 12, and ≥40 days, respectively). In contrast, HER2 knockdown was less effective in inhibiting growth of trastuzumab‐resistant JIMT‐1 cells in vitro and in vivo. Combined administration of trastuzumab and radiation treatment was also analyzed using T47D 3D microtissues. Administration of both, radiation (5 Gy) and trastuzumab, significantly enhanced the growth inhibiting effect in 3D microtissues. To improve the predictive power of potential drugs—as single agents or in combination—here, we show that regarding tumor growth analyses, 3D microtissues are highly comparable to outcomes derived from xenografts. Considering increased limitations for animal experiments on the one hand and strong need of novel drugs on the other hand, it is indispensable to include highly reproducible 3D microtissue platform in preclinical analyses to validate more accurately the capacity of future drug‐combined radiotherapy.

## Introduction

Proliferation assays of two‐dimensional (2D) monolayer cancer cells are too artificial for anticancer drug screening and fail to model three‐dimensional (3D) solid tumor 1, 2. Meanwhile, the limitations of 2D models are considered as one major reason that around 95% of potential anticancer drugs fail in clinical trials although initially showing high antitumor activity in vitro 3. Multicellular 3D spheroid models have been proven to be more physiologically relevant to in vivo tumors. Regarding cancer research, Sutherland and colleagues pioneered in 3D cell culture model generating Chinese hamster lung spheroids in rotary flasks 4. Since then, various systems have been developed including spontaneous aggregation in drops 5, 6, spinner flasks 7, and scaffold‐based systems 8. 3D models can help investigating the interplay between different physiological conditions (oxygen or nutrient deprivation), irradiation or other physical and chemical stimuli 9, 10. Additionally, they allow for long‐term studies of several weeks 9, 11, 12. Nevertheless, further studies are needed to verify that 3D models can mimic in vivo tumors.

We focused on the therapeutically relevant oncogene HER2 (human epidermal growth factor receptor 2) regulating mammary gland tumorigenesis 13, 14. HER2 overexpression occurs in approximately 30% of breast tumors and is associated with malignancy and a poor prognosis 15. In 1998, the antibody‐based targeted therapy for HER2‐positive tumors using trastuzumab has shown a survival benefit 16. Here, the growth rates of HER2‐depleted trastuzumab‐sensitive T47D cells and trastuzumab‐resistant JIMT‐1 cells were analyzed in 2D monolayer cultures, 3D microtissues and in xenografts. To improve HER2‐targeted therapy, we treated T47D microtissues with trastuzumab combined with radiation in 2D and 3D.

## Materials and Methods

### 2D monolayer cultivation and stable knockdowns

The trastuzumab‐sensitive T47D and the trastuzumab‐resistant JIMT‐1 breast cancer cell lines were used. The T47D cells (HTB‐133) were acquired from the American Type Culture Collection and were maintained in RPMI 1640 with GlutaMAX (Roswell Park Memorial Institute, Life Technologies GmbH, Darmstadt, Germany). The JIMT‐1 cells (ACC‐589) were acquired from the German Collection of Microorganisms and Cell Cultures (Heidelberg, Germany) and were maintained in DMEM (Dulbecco′s modified eagles medium) with GlutaMAX. Both media were supplemented with 10% fetal bovine serum (both from Life Technologies GmbH) and with human insulin (10 *μ*g/mL, Sigma, St. Louis, MO) and the cells were incubated at 37°C in 5% CO_2_. Two independent infections with lentiviral particles using LentiBoost adjuvant (Sirion Biotech GmbH, Martinsried, Germany) were conducted as described 14, 17, 18.

### Cell proliferation assays

Cell proliferation was analyzed using water‐soluble tetrazolium 1 (WST‐1) in a colorimetric assay in quadruplicates (Roche Diagnostics, Mannheim, Germany) or using CellTiterGlo Luciferase assay (Promega, Madison, WI) according to the manufacturers' protocol.

### 3D microtissues and treatments

The breast cancer cells were seeded with 500 cells per drop into scaffold‐free 96‐well InSphero culture GravityPLUS^™^ plates (InSphero AG, Schlieren, Switzerland). 3D microtissues were produced within 3 days and transferred into InSphero GravityTRAP^™^ plates. Growth of six spheroids per approach was analyzed every 3 days by the high content screening system Operetta (Perkin Elmer, Waltham, MA) and quantified in maximal area of GFP‐expressing microtissue (*μ*m^2^) using the Harmony analysis Software (Perkin Elmer) 19. Medium was refreshed at day 6 posttransfer to assay plates. T47D monolayers and 3D microtissues were irradiated with a Cs‐137 irradiator (HWM D‐2000, Siemens, Erlangen, Germany) at 0.95 Gy/minute. A dose of 5 Gy at room temperature was administered and control monolayers and microtissues were sham irradiated. After 30 min, trastuzumab (10 *μ*g/mL, Roche Diagnostics) was added when appropriate.

### Immunofluorescence of 3D microtissue sections

Six microtissues were pooled in PBS at indicated time points and fixed for 1 h in 4% PBS‐buffered paraformaldehyde. 50 *μ*L of human plasma were mixed with the microtissues, 50 *μ*L of thrombin (1000 U/mL, both Sigma) were pipetted in the lid of an Eppendorf tube. By centrifugation at 300 g for 10 min the fluids clot. The microtissue clot was placed in a tissue cassette, processed in a vacuum Tissue‐Tek VIP 6 device (Sakura, Torrance, CA) and embedded in paraffin. Sections of 4 *μ*m were cut from each paraffin‐embedded microtissue block and dried on glass slides. For staining, sections were deparaffinized and one section was stained with hematoxylin and eosin (H&E, Carl Roth, Karlsruhe, Germany). Sections of 3D microtissues grown for 12 days were incubated with a primary antibody against Ki67 for proliferative cells (clone SP6, Novus Biologicals, Littleton, CO) or with a primary antibody against cleaved Caspase‐3 specific for apoptotic cells (Abcam, Cambridge, UK) and analyzed by fluorescence microscopy using anti‐rabbit Alexa488‐ or Cy3‐conjugated secondary antibodies (Life Technologies GmbH). Nuclei were visualized with DAPI (Vectashield, Vector Laboratories Inc, Burlingame, CA). Appropriate negative controls were obtained.

### In vivo xenograft model

The animal studies were performed in accordance with the German and European laws on animal welfare. A total of 10^6^ cells were orthotopically injected into female mice as described 14. Tumor sizes in mm^2^ area were quantified from at least six animals per group once a week for up to 6 weeks.

### Western blots of cryopreserved and FFPE xenograft samples

Since a pool of sections per sample was necessary to extract proteins, only JIMT‐1 xenografts ≥1 cm^3^ were used, due to sufficient xenograft material for extractions and analysis (Fig. S2A). For protein extraction from cryopreserved xenografts, 20 × 20 *μ*m thick sections were lysed in 150 *μ*L TPER buffer and sonicated (Sonoplus; Bandelin, Berlin, Germany). For protein isolation from FFPE xenografts, a minimum of 5 × 20 *μ*m sections were lysed as previously described 20 with minor modifications: the sections were deparaffinized, rehydrated, washed in 0.5% *β*‐d‐octylglucopyranosid and lysed in TPER protein isolation buffer additionally containing 20 mmol/L Tris‐HCl pH 8.8, 2% SDS, 1% *β*‐d‐octylglucopyranosid, 200 mmol/L glycine. The samples were incubated for 20 min at 95°C, followed by 2 h at 80°C and sonicated. The suspensions were centrifuged, the supernatants were applied for immunoblotting as described 14 using the following antibodies: anti‐HER2 (Dako, Glostrup, Denmark) and anti‐Tubulin (Sigma‐Aldrich, Taufkirchen, Germany).

### Statistics

For statistical analysis Student's *t*‐test was used (SigmaPlot, Systat Software GmbH, Erkrath, Germany) and statistical significance was considered at *P* < 0.05.

## Results

### Growth analysis of tumor cells cultivated as 3D microtissues is more comparable to in vivo xenografts than to outcomes of 2D monolayers

**Figure 1:**
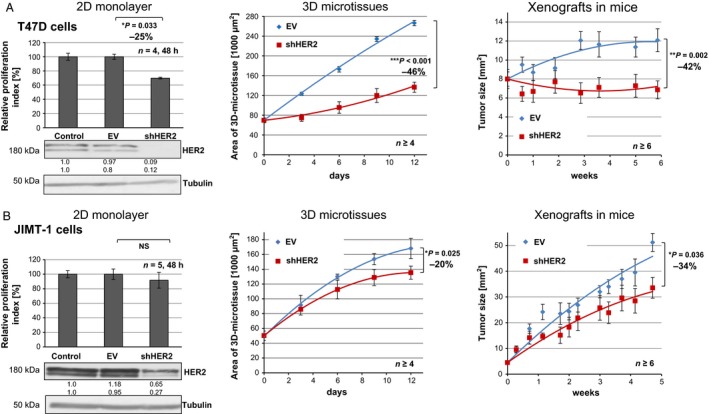
Comparison of growth rates after human epidermal growth factor receptor 2 (HER2) downregulation using 2D monolayers, 3D microtissues and in vivo xenografts reveals 3D and in vivo models as more comparable and significant than 2D monolayers. T47D cells (A) and JIMT‐1 cells (B) lentivirally transduced with a GFP‐encoding control empty vector (EV, blue) or shHER2 knockdown vector (shHER2, red) were cultured in 2D monolayers (quantified with CellTiterGlo cell proliferation assay after 48 h, *n* = 4, *left*; the band intensities were quantified in relation to control as described 14), the mean values of two independent infections are shown, 3D microtissues (quantified using GFP area determination over 12 days, *n* ≥ 4, *middle*) and in vivo xenografts grown in nude mice (quantified via calipers over 5–6 weeks, *n* ≥ 6, *right*) (*P < 0.05, **P < 0.01, ***P < 0.001, NS ‐ not significant).

To compare short‐ with long‐term analysis of growth rates, T47D and JIMT‐1 cells were cultured in 2D, 3D or as xenografts (Figs. 1 and S1A,C). Breast cancer cells were transduced with a GFP‐encoding empty vector control (EV) or a HER2 knockdown vector (shHER2). In 2D, HER2‐depleted T47D breast cancer cells (−90% of HER2 protein expression, Fig. 1A, left down), showed reduced cell proliferation compared with nontransduced (control) and empty vector transduced cells (Fig. 1A, left up). Although T47D cells moderately express HER2 21, its knockdown significantly reduced cell proliferation (−25%; *P* < 0.033). In trastuzumab‐resistant JIMT‐1 cells, knockdown of HER2 reduces protein expression (−35% or −70%, Fig. 1B, left down), but cell proliferation in 2D was not significantly altered (−10%; Fig. 1B, left up).

Remarkably, in 3D, compared with control empty vector, HER2‐depleted T47D microtissue growth was strongly and significantly reduced (−46%; *P* < 0.001, Fig. 1A). Considering that the same tumor cells were applied for 2D and 3D assays, this result demonstrates that the effect on proliferation in 3D was more prominent than in 2D. Regarding JIMT‐1 cell growth in 3D, the HER2 knockdown led to a significant growth reduction of approximately 20% compared with control empty vector (Fig. 1B, *P* = 0.025) indicating a stronger effect in 3D. In T47D cells (Fig. S1B) and in JIMT‐1 cells (Fig. S1D), the HER2 RNA expression was reduced to approximately 35% (after 12 days), demonstrating efficient and stable HER2 lentiviral knockdown in both breast cancer cell lines.

Xenografts were generated either using T47D or JIMT‐1 transduced cells. Control tumor xenografts differed in absolute tumor sizes as JIMT‐1 xenografts developed more tumor mass (Fig. S2A). Compared with T47D controls (EV), HER2‐depleted xenografts show a significantly reduced size (−42%, *P* = 0.002, Fig. 1A, right). JIMT‐1 control xenografts (EV) compared with HER2‐depleted xenografts (−50% HER2 protein expression, Fig. S2B, right) also exhibit a significantly reduced size (−34%, *P* = 0.036, Fig. 1B, right). In summary, two differentially HER2‐expressing breast cancer cell lines were analyzed in 2D monolayers, 3D microtissues, and xenografts for effects on proliferation following stable HER2 knockdown. In all three models, HER2 knockdown affected the proliferation of T47D cells more than of JIMT‐1 cells. Interestingly, the impact was stronger, more considerable and more similar in 3D models (up to 12 days) and xenografts (up to 40 days) than observed in 2D (2 days).

### T47D cells form functional tumor tissue‐like 3D microtissues

**Figure 2:**
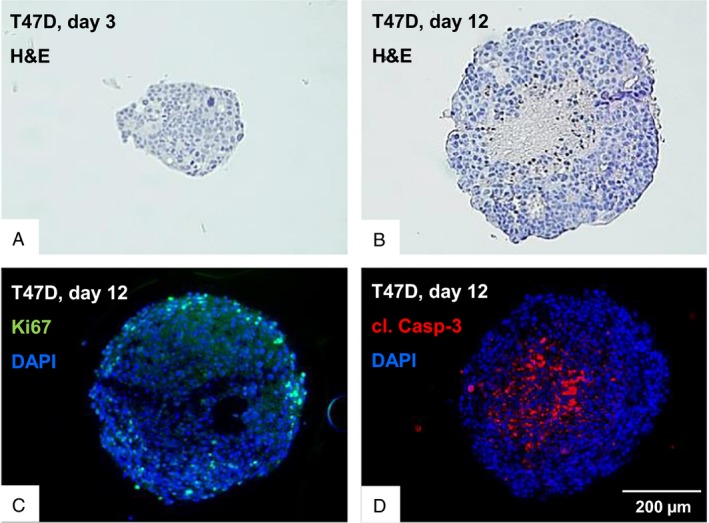
T47D cells form functional 3D spheroids in a scaffold‐free system. Upper line images show H&E stainings of microtissues grown for 3 days (A) and 12 days (B) after the drop in a GravityTRAP ^™^ plate. In addition, sections of 3D microtissues grown for 12 days were incubated with DAPI and an antibody against Ki67 (green) to stain proliferative cells (C) or with an antibody against cleaved Caspase‐3 (red) specific to stain apoptotic cells (D) and analyzed by fluorescence microscopy. Nuclei were visualized with DAPI (blue).

A scaffold‐free system for 3D spheroid growth in a hanging drop was adapted for immunofluorescence analysis (Fig. 2). Middle parts of T47D 3D microtissues after 3 days growth show dense but homogenous cell assembly (Fig. 2A). Nine days later (Fig. 2B), spheroids display a gradient of Ki67‐positive proliferating cells located in the outer cell layers (Fig. 2C), whereas cleaved Caspase‐3‐positive apoptotic cells are located in the core region (Fig. 2D). These variations to physiological nutrient and oxygen gradients resemble changes in early neoplasia.

### Additive growth inhibition is detected using T47D 3D microtissues followed by HER2 knockdown or anti‐HER2 treatment combined with irradiation

**Figure 3:**
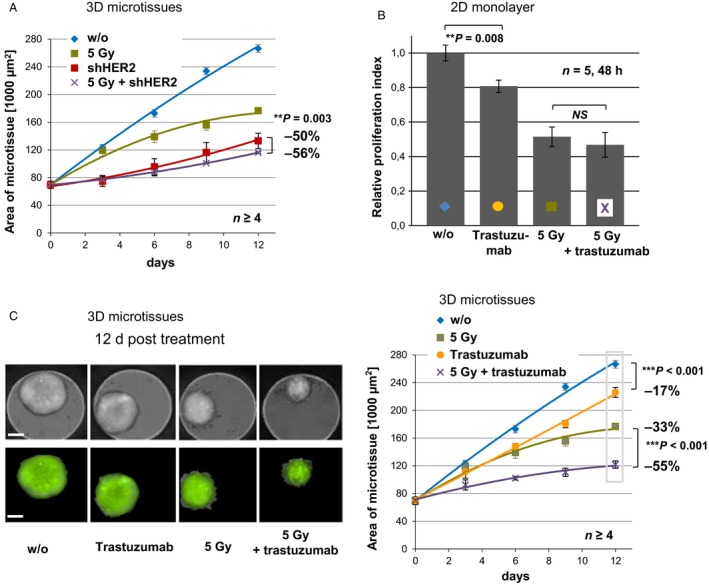
Additive growth inhibition of T47D cells following knockdown of HER2 or anti‐HER2 treatment combined with irradiation is significant in 3D and not in 2D. (A) Growth of T47D 3D microtissues without (w/o, blue) or after HER2 downregulation (shHER2, red) was followed up to 12 days in combination with a single dose of radiation at day 0 (5 Gy; green and purple). (B) T47D cells (2D monolayers) were analyzed without (w/o, blue) or after single (yellow) or combined (purple) treatment with the anti‐HER2 antibody trastuzumab (10 *μ*g/mL) and radiation (5 Gy, green). Cell proliferation was quantified by WST‐1 cell proliferation assay after 48 h (*n* = 5). (C) Representative captions of T47D 3D microtissues without treatment (w/o) or after single or combined treatment with trastuzumab (10 *μ*g/mL) and radiation (5 Gy). Microscopic analysis was performed using the Operetta screening system (*left*, scale bar = 200 *μ*m). T47D 3D microtissue growth was quantified using GFP area determination over 12 days (*n* ≥ 4, *right*) (**P < 0.01, ***P < 0.001, NS ‐ not significant).

T47D cells grown in 3D microtissues or as xenografts have demonstrated substantial growth reduction following HER2 downregulation. To evaluate therapeutic interventions based on HER2 signaling in HER2‐dependent cells, we treated control cells and HER2‐depleted T47D 3D microtissues with a single dose of irradiation (5 Gy, Fig. 3A). Though downregulation of HER2 has a very potent effect on 3D microtissue growth (−50% vs. w/o, Fig. 3A), the combination with irradiation led to further significant inhibition compared to HER2 knockdown alone (−56% vs. w/o, *P* = 0.003, Fig. 3A).

In 2D and 3D, trastuzumab inhibited T47D cell growth (Fig. 3B, *P* = 0.008; Fig. 3C, right, *P* < 0.001), whereas growth of JIMT‐1 3D microtissues was not altered in the presence of trastuzumab (Fig. S1E). Trastuzumab combined with radiation using T47D cells cultured in 2D showed a minor inhibitory effect (Fig. 3B). Remarkably, in 3D microtissues, additive reduced growth following radiation with combined trastuzumab treatment was observed (−55% vs. −33% (5 Gy) or −17% (trastuzumab), Fig. 3C, *P* < 0.001). In summary, T47D cells in 2D and 3D models were exposed to radiation and trastuzumab to demonstrate combination treatments as more powerful than single treatment regarding cancer therapies. Here, an additional and statistically significant inhibition following the combined treatment was observed using the physiologically more relevant 3D model.

## Discussion

Since HER2 is therapeutically used in breast cancer 14, we applied a novel technique to culture tumor cells as 3D microtissues. Presented data demonstrate the feasibility of 3D microtissues for efficient drug evaluation and validation in preclinical analyses. Additionally, there is an emerging body of evidence showing that, for example, proliferation inhibitors differently affect 3D models compared to 2D monolayers. Significant anti‐proliferative effects of the PI3K‐inhibitor PX‐866 were observed in spheroids derived from several cancer cells, whereas there was no effect on these cells grown as monolayers 21. Furthermore, a greater tumor proliferation inhibition was demonstrated when T47D 3D spheroids were exposed to trastuzumab in contrast to 2D models 22. Recent paper by Rodriguez et al. describes that 3D organization could decrease trastuzumab sensitivity of HER2 overexpressing cells compared to monolayer cell culture. Probably, the changed organization of cells grown in 2D or 3D system may lead to different outcomes due to changed access of nutrients, oxygen, or drugs 3, 9.

In this study, we have compared 2D and 3D assays using JIMT‐1 trastuzumab‐resistant cells 14, 23. In contrast to JIMT‐1 cells, T47D cells do respond to anti‐HER2 trastuzumab treatment 14. In preclinical studies, combining radiotherapy with trastuzumab has been described as beneficial because trastuzumab elevates radiosensitivity by oxygenating tumor tissue 22. Combined HER2‐targeted immunotherapy using trastuzumab with radiotherapy has been considered to potentially decrease mammary tumor growth in vitro and in vivo 24. In addition, radiation can increase expression levels of HER2 as target antigen of trastuzumab 24, 25. Therefore, applying trastuzumab treatment before or concurrent with irradiation could potentially further increase observed radiosensitizing effect.

Several studies including different 3D systems were applied 26, 27 however, little is known regarding 3D breast cancer cell spheroids exposed to radiation although radiotherapy is widely applied for breast cancer therapy 28.

Cultivating 3D microtissues using a hanging drop technology guarantees minor deviations within replicates 6, 19 and allows easy handling regarding single or combined radiation and drug treatment. Starting with only 500 tumor cells per hanging drop, 12 days follow‐up allows “long‐term” investigations, comparisons of immediate, mediate and late effects of oncogene knockdown and further analysis of combined treatments. To demonstrate the power of 3D microtissues for combination therapies, we treated T47D cells with trastuzumab and irradiation in 2D and 3D models. We observed remarkable additive proliferation inhibition only in 3D microtissues enabling long‐term analysis and better correlation with native tumor architecture.

Meanwhile, the numerous advantages of such 3D systems dominate and have the potential to predict therapeutic windows including combined treatments more accurately. Considering increased limitations for animal experiments but increasing need of novel drugs, usage of highly reproducible 3D models is indispensable. Subsequent in vivo studies will benefit from smaller cohort sizes saving animals and general costs. Instead of injecting millions gel‐embedded cells from cell lines or tumors, precultured 3D microtissues can be pooled and injected orthotopically. Higher engraftment rates of these 3D cultured cells and minor deviations in xenograft growth have been shown; both factors that improve the performance of in vivo studies 29. Furthermore, ex vivo approaches using tumor biopsies may be cultured using such 3D system 3 and then treated with therapeutically relevant compounds and radiation to predict the tumor response to the combined treatment. Studying in vitro long‐term effects of potential therapeutics as single agents or in combination, 3D microtissues essentially contribute to a more accurate tool predicting tumor growth and therapeutic outcomes.

## Conflict of Interest

All authors declare that they have no competing interests.

## Supporting information


**Figure S1.** T47D and JIMT‐1 3D microtissue analyses after HER2 downregulation. (A, C) Example of GFP detection for 3D microtissues generated from T47D (A) and JIMT‐1 cells (C) at day 3 (*upper line*) and day 12 (*lower line*) using an empty vector control encoding GFP (EV) and a HER2‐downregulating vector (shHER2). (B, D) Relative HER2 mRNA expression in T47D (B) and JIMT‐1 (D) 3D microtissues lentivirally transduced with a GFP‐encoding control vector (EV) or a HER2‐downregulating vector (shHER2) and grown for 12 days. For quantitative reverse transcription PCR (qRT‐PCR), 6 T47D or JIMT‐1 3D microtissues grown for 12 days with and without treatment were pooled and RNA was isolated using phenol/chloroform buffer peqGOLD TriFast (Peqlab, Erlangen, DE) followed by automated purification using a Maxwell16 device according to manufacturer's instructions (Promega, Madison, WI). RNA was converted to cDNA by a reverse transcription kit (QuantiTect by Qiagen, Hilden, Germany) and quantified by TaqMan gene expression assays for HER2 (Hs01001580_m1) and TBP as internal control (Hs00427620_m1) using the StepOne RT‐PCR System following the manufacturer's instructions (Life Technologies). (E) JIMT‐1 3D microtissues were analyzed without (w/o, blue) or after single (yellow) treatment with the anti‐HER2 antibody trastuzumab (10 *μ*g/mL). 3D microtissue growth was quantified using GFP area determination over 12 days (*n* ≥ 4).

**Figure S2.** T47D and JIMT‐1 xenografts analyses after HER2 knockdown. (A) Representative examples of in vivo xenografts 6 weeks (T47D) and 5 weeks (JIMT‐1) post inoculation (p.i.) after extraction, formalin fixation and paraffin embedding (FFPE). (B) Western blot analysis of FFPE protein extracts from xenografts derived from JIMT‐1 cells lentivirally transduced with a GFP‐encoding control vector (EV) or a HER2 knockdown vector (shHER2).
