# Supplementary material for: Three‐dimensional microtissues essentially contribute to preclinical validations of therapeutic targets in breast cancer
Source: Cancer Med. 2016 Jan 14;5(4):703–10. doi: 10.1002/cam4.630 (PMC4831289; doi:10.1002/cam4.630)
Supplement: Supplementary file 1 — Figure S1. T47D and JIMT‐1 3D microtissue analyses after HER2 downregulation. (A, C) Example of GFP detection for 3D microtissues generated from T47D (A) and JIMT‐1 cells (C) at day 3 (upper line) and day 12 (lower line) using an empty vector control encoding GFP (EV) and a HER2‐downregulating vector (shHER2). (B, D) Relative HER2 mRNA expression in T47D (B) and JIMT‐1 (D) 3D microtissues lentivirally transduced with a GFP‐encoding control vector (EV) or a HER2‐downregulating vector (shHER2) and grown for 12 days. For quantitative reverse transcription PCR (qRT‐PCR), 6 T47D or JIMT‐1 3D microtissues grown for 12 days with and without treatment were pooled and RNA was isolated using phenol/chloroform buffer peqGOLD TriFast (Peqlab, Erlangen, DE) followed by automated purification using a Maxwell16 device according to manufacturer's instructions (Promega, Madison, WI). RNA was converted to cDNA by a reverse transcription kit (QuantiTect by Qiagen, Hilden, Germany) and quantified by TaqMan gene expression assays for HER2 (Hs01001580_m1) and TBP as internal control (Hs00427620_m1) using the StepOne RT‐PCR System following the manufacturer's instructions (Life Technologies). (E) JIMT‐1 3D microtissues were analyzed without (w/o, blue) or after single (yellow) treatment with the anti‐HER2 antibody trastuzumab (10 μg/mL). 3D microtissue growth was quantified using GFP area determination over 12 days (n ≥ 4). [file CAM4-5-703-s001.pdf]

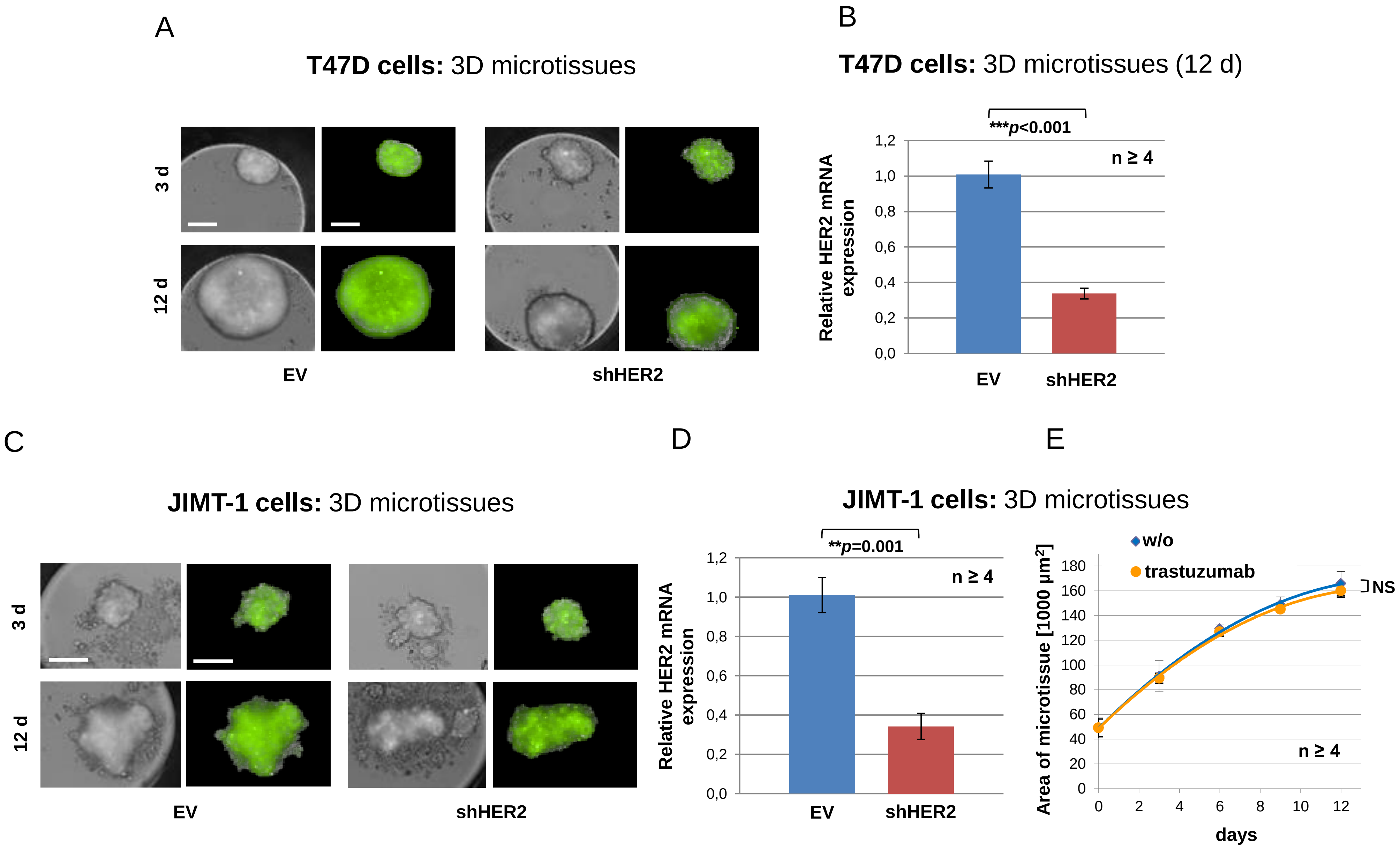
A
T47D cells: 3D microtissues
3 d
12 d
EV
shHER2
B
T47D cells: 3D microtissues (12 d)
***p<0.001
n ≥ 4
Relative HER2 mRNA expression
1,2
1,0
0,8
0,6
0,4
0,2
0,0
EV
shHER2
C
JIMT-1 cells: 3D microtissues
3 d
12 d
EV
shHER2
D
E
JIMT-1 cells: 3D microtissues
**p=0.001
n ≥ 4
Relative HER2 mRNA expression
1,2
1,0
0,8
0,6
0,4
0,2
0,0
EV
shHER2
w/o
trastuzumab
NS
n ≥ 4
Area of microtissue [1000 µm²]
180
160
140
120
100
80
60
40
20
0
0
2
4
6
8
10
12
days

Suppl. Fig. 1
