# Supplementary figures and images for: Three‐dimensional microtissues essentially contribute to preclinical validations of therapeutic targets in breast cancer
Source: Cancer Med. 2016 Jan 14;5(4):703–10. doi: 10.1002/cam4.630 (PMC4831289; doi:10.1002/cam4.630)

A

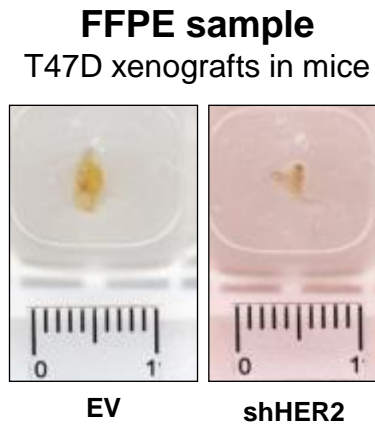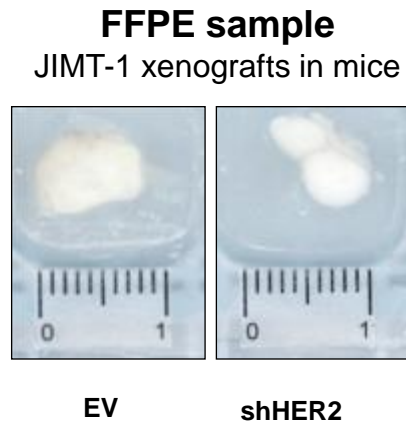

B

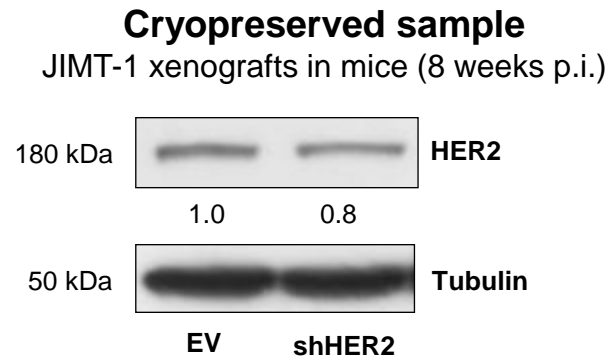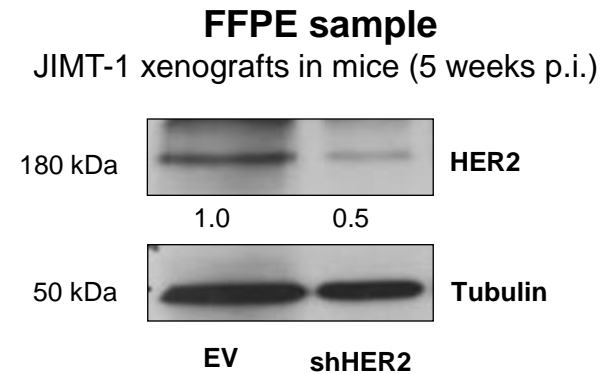

Supplement: Supplementary file 2 — Figure S2. T47D and JIMT‐1 xenografts analyses after HER2 knockdown. (A) Representative examples of in vivo xenografts 6 weeks (T47D) and 5 weeks (JIMT‐1) post inoculation (p.i.) after extraction, formalin fixation and paraffin embedding (FFPE). (B) Western blot analysis of FFPE protein extracts from xenografts derived from JIMT‐1 cells lentivirally transduced with a GFP‐encoding control vector (EV) or a HER2 knockdown vector (shHER2). [file CAM4-5-703-s002.pdf]
